# Characterization of Dynamic Behaviour of MCF7 and MCF10A Cells in Ultrasonic Field Using Modal and Harmonic Analyses

**DOI:** 10.1371/journal.pone.0134999

**Published:** 2015-08-04

**Authors:** Annette Geltmeier, Beate Rinner, Dennis Bade, Katharina Meditz, Reiner Witt, Uwe Bicker, Catrin Bludszuweit-Philipp, Patrick Maier

**Affiliations:** 1 ASD Advanced Simulation & Design GmbH, Rostock, Germany; 2 Center for Medical Research, Medical University Graz, Graz, Austria; 3 Mechatronic AG, Darmstadt, Germany; 4 Oncowave Medical AG, Darmstadt, Germany; 5 Department of Radiation Oncology, Universitätsmedizin Mannheim, Medical Faculty Mannheim, Heidelberg University, Mannheim, Germany; Taipei Medicine University, TAIWAN

## Abstract

Treatment options specifically targeting tumour cells are urgently needed in order to reduce the side effects accompanied by chemo- or radiotherapy. Differences in subcellular structure between tumour and normal cells determine their specific elasticity. These structural differences can be utilised by low-frequency ultrasound in order to specifically induce cytotoxicity of tumour cells. For further evaluation, we combined *in silico* FEM (finite element method) analyses and *in vitro* assays to bolster the significance of low-frequency ultrasound for tumour treatment. FEM simulations were able to calculate the first resonance frequency of MCF7 breast tumour cells at 21 kHz in contrast to 34 kHz for the MCF10A normal breast cells, which was due to the higher elasticity and larger size of MCF7 cells. For experimental validation of the *in silico*-determined resonance frequencies, equipment for ultrasonic irradiation with distinct frequencies was constructed. Differences for both cell lines in their response to low-frequent ultrasonic treatment were corroborated in 2D and in 3D cell culture assays. Treatment with ~ 24.5 kHz induced the death of MCF7 cells and MDA-MB-231 metastases cells possessing a similar elasticity; frequencies of > 29 kHz resulted in cytotoxicity of MCF10A. Fractionated treatments by ultrasonic irradiation of suspension myeloid HL60 cells resulted in a significant decrease of viable cells, mostly significant after threefold irradiation in intervals of 3 h. Most importantly in regard to a clinical application, combined ultrasonic treatment and chemotherapy with paclitaxel showed a significantly increased killing of MCF7 cells compared to both monotherapies. In summary, we were able to determine for the first time for different tumour cell lines a specific frequency of low-intensity ultrasound for induction of cell ablation. The cytotoxic effect of ultrasonic irradiation could be increased by either fractionated treatment or in combination with chemotherapy. Thus, our results will open new perspectives in tumour treatment.

## Introduction

Clinical tumour treatment by radio- or chemotherapy is often accompanied by severe side effects since both methods also induce damage of normal tissue cells. Thus, further treatment options that target the tumour cells more specifically are urgently needed. In this regard, the differences in subcellular structure between tumour and normal cells might be utilised for induction of selective cytotoxicity [1]. These structural differences determine the specific elasticity of whole cells [2] or of subcellular structures described by the Young’s modulus. Through various methods like micropipette aspiration [1], optical tweezers [3], or acoustic force microscopy [4], it was shown that single cells of various tumour entities were about twofold softer than corresponding normal tissue cells [5]. Differences in the size of the actin cortex attached to the cellular membrane, the composition of the cytoskeleton (mostly due to various amounts of intermediate filament proteins), and the nuclear lamin network are responsible for these discrepancies in elasticity between normal and tumour cells [6].

Thus, a method utilising the differences in (sub-)cellular elasticity to specifically induce cytotoxicity of tumour cells would open new perspectives in tumour treatment. In the recent years, irradiation with low-frequency ultrasound was shown to produce biologic alterations in tumour cells [7–11] even with distinct sensitivity in normal cells [12, 13]. However, the understanding remains rudimentary about which part of a cell contributes to its overall elasticity and is affected by low-frequency ultrasound and is thus responsible for induction of cell death. In order to further elucidate and simulate the behaviour of a cell and its components like nucleus or cytoskeleton irradiated with ultrasound, an FEM (finite element method) analysis presents an adequate method that might also enable the natural frequencies specific to various cell types to be determined [14–17]. The same methods used to determine cellular elasticity were also applied for the evaluation of additional mechanical properties of single cells and isolated nuclei [15, 18, 19] and were in some cases accompanied by numerical simulations [15, 18].

In an exemplary analysis, a linear model was generated for a spherical object, representing a typical organelle such as the nucleus, within a homogenous viscoelastic medium that vibrates uniformly [20]. The structure in which the object is embedded was described herein by four different rheological models. Resonance frequency with maximum amplitudes of intracellular structure was found in this analysis to lie between tens and hundreds of kHz. In another *in silico* approach, the modelled natural frequency of the cytoskeleton as the frequency for induction of cell collapse and death was significantly lower for cancer cells in contrast to normal cells (131 vs. 415 MHz) suggesting the possibility of selective cytotoxicity [21]. For theoretical determination of natural frequencies of the membrane and the cytoplasm of bacterial cells, a shell model was developed to determine the motion of the cell in an ultrasonic field by the motion of the internal viscous fluid, a thin elastic shell, and the surrounding viscous fluid [22, 23]. Dynamic modelling and FEM analysis were used to determine the Young’s modulus of the cell wall of yeast cells using their known resonance frequency [24]. The method of frequency response (dynamic compression and recovery) using a piezoelectric actuator which excites a single cell in sinusoidal fashion was suggested as a new physical marker to differentiate the human breast cancer MCF7 cells from normal MCF10A human breast cells [25, 26]. Frequency and preload-dependent differences were found in the deformability of both cell types. Both cell lines were ideally suited for prediction of dynamic behaviour within the ultrasonic field and a possible distinction between both cell lines, since detailed analysis of the appropriate cellular properties has been performed in recent years.

For our FEM analysis, we used data from AFM (atomic force microscopy) tests on MCF7 and MCF10A cells for the properties of cellular components [19]. Further important values for cell modelling, like diameter, shape and volume of cells and nuclei of benign (MCF10A) and cancerous (MCF7) human breast epithelial cells were also derived from literature [5, 15, 18, 19, 27–29] or additionally determined by using a CASY cell counter (see Table 1 and references therein).

**Table 1:** 

parameter / unit	MCF7	MCF10A
cell volume / μm^3^	3375–16873 ^a^	678–1317 ^a^
hydrodynamic mass / kg ^b^	1.74–7.34 x 10^−12^	0.69–3.43 x 10^−12^
actin cortex diameter / μm	1.27–2.17 (13.5%) [14, 45]	1.37–2.47 (20%) [14, 45]
actin cortex density / g/cm^3^	1.15 [14]	1.15 [14]
actin cortex E-modulus / kPa	1.125 [45]	1.1 [45]
actin cortex Poisson‘s ratio	0.4999 [53]	0.4999 [53]
cytoplasm density / g/cm^3^	1.05 ^c^	1.05 ^c^
cytoplasm Young’s modulus / kPa	0.15 or 0.47	0.25 or 0.7
cytoplasm Poisson‘s ratio	0.4999 [53]	0.4999 [53]
nucleus volume / μm^3^	1122–1572 [27]	360–653 [29]
nucleus eccentricity / μm	1.35–1.65 [29]	1.97–2.21 [29]
nucleus density / g/cm^3^	1.3	1.3
nucleus Young’s modulus / kPa	0.15[28] or 4.7 [14, 15, 30]	0.25 [28] or 7 [14, 15, 30]
nucleus Poisson’s ratio	0.4999 [53]	0.4999 [53]

^a^ determined by measurements of 10 000 cells using a CASY Cell Count (Merck, Darmstadt, Germany)

^b^ approximately calculated as the hydrodynamic mass of a sphere whose radius is obtained from the average of the radii of the different cell sizes

^c^ calculated with cytosol as an aqueous solution (density of 1 g/cm^3^) containing a proportion of proteins of 15% with an average density of 1.7 g/cm^3^ [54, 55]

In a first step of the analyses, the material components were validated in an FEM model. The second part included a modal analysis of single cells to determine the natural frequencies of MCF7 and MCF10A cells. A parameter study of the biological range of cell dimensions and material behaviour was performed to show the influence of geometric and material properties on the natural frequencies. In a third part, harmonic vibration analysis was performed for single cell models under oscillating hydrostatic pressure from the ultrasonic field. The determination of resonance frequencies and the corresponding amplitudes were used to predict scenarios of possible damage of the cells in an ultrasonic field. Finally, the *in silico*-generated results of the numerical analysis were validated in various *in vitro* 2D and 3D experimental settings with treatment of MCF7 and MCF10A cells with low-frequency ultrasound.

## Results

### 1 Validation

**Fig 1:**
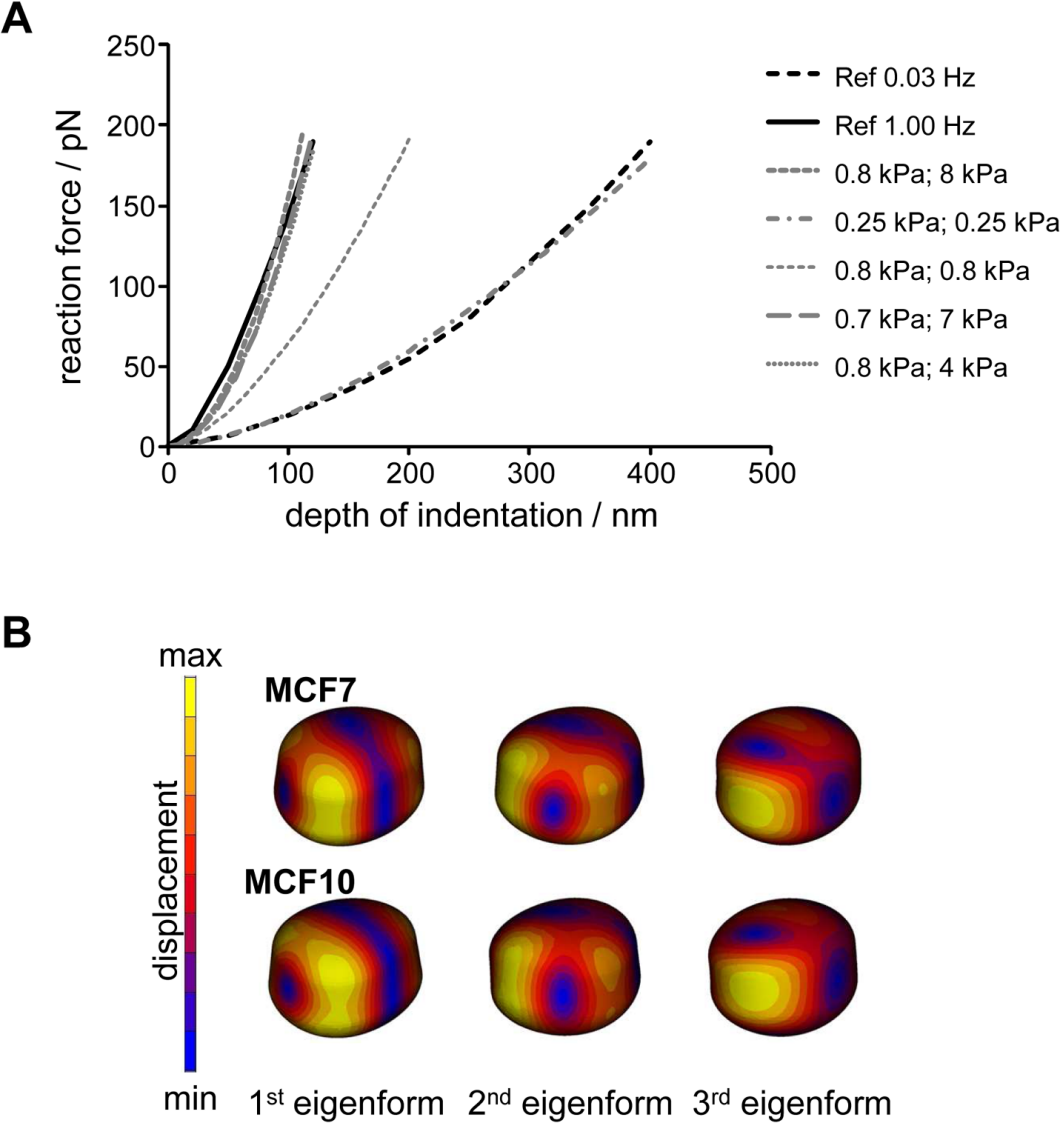
Depth of indentation for MCF10A cells in water. (A). Calculated reaction force–deformation curves for different parameter sets for Young’s modulus of cytoplasm and nucleus (first and second value in parenthesis) for cell type MCF10A compared to reference curves from the literature (Li et al, 2008). (B) Displacements of the first three eigenforms for MCF7 and MCF10A cells in water.

From the FEM analysis simulating the AFM experiments reported in the literature (Li et al, 2008), validation of the assumed cell material parameters was obtained. In this sense, the reaction force-deformation relationship for the different material parameter combinations for nucleus and cytoplasm were calculated from the FE results and compared with the reference curves determined experimentally for the lowest and highest AFM loading rates (1 Hz and 0.03 Hz, respectively) (Fig 1A). Young’s modulus reference values for cytoplasm, and nucleus for the modal analysis were chosen from the best possible match of the validation simulations with the experimental curve at an AFM loading rate of 1Hz. These were 0.7 kPa and 7 kPa for MCF10A cells and 0.47 kPa and 4.7 kPa for MCF7 cells, respectively. The minimum elasticity values for cytoplasm and nucleus to be used in the modal analysis were defined from fitting with the minimal experimental loading rate of 0.03 Hz. For both compartments, 0.25 kPa were calculated for MCF10 cells and 0.15 kPa for MCF7 cells.

### 2 Modal analysis

In the next step, a modal analysis was performed using reference values for the Young’s modulus of cytoplasm and nucleus which were obtained from the above-mentioned validation studies. The modal analysis was used to characterize the eigenoscillation behaviour of the structure without external excitation. The identified mode shapes and natural frequencies provide evidence regarding what form and with which frequency the structure oscillates freely on the sole basis of its mass and stiffness as well as under defined fixation conditions. The modal analysis was used to predict the first natural frequencies and eigenforms of MCF7 and MCF10A cells for a large number of variations with respect to geometry, material and boundary conditions. An example of the shape of the deformed cells for the first three modes is shown in Fig 1B comparing MCF-7 and MCF10A cells. Slight differences could be observed between both cell types in the cell deformations.

**Fig 2:**
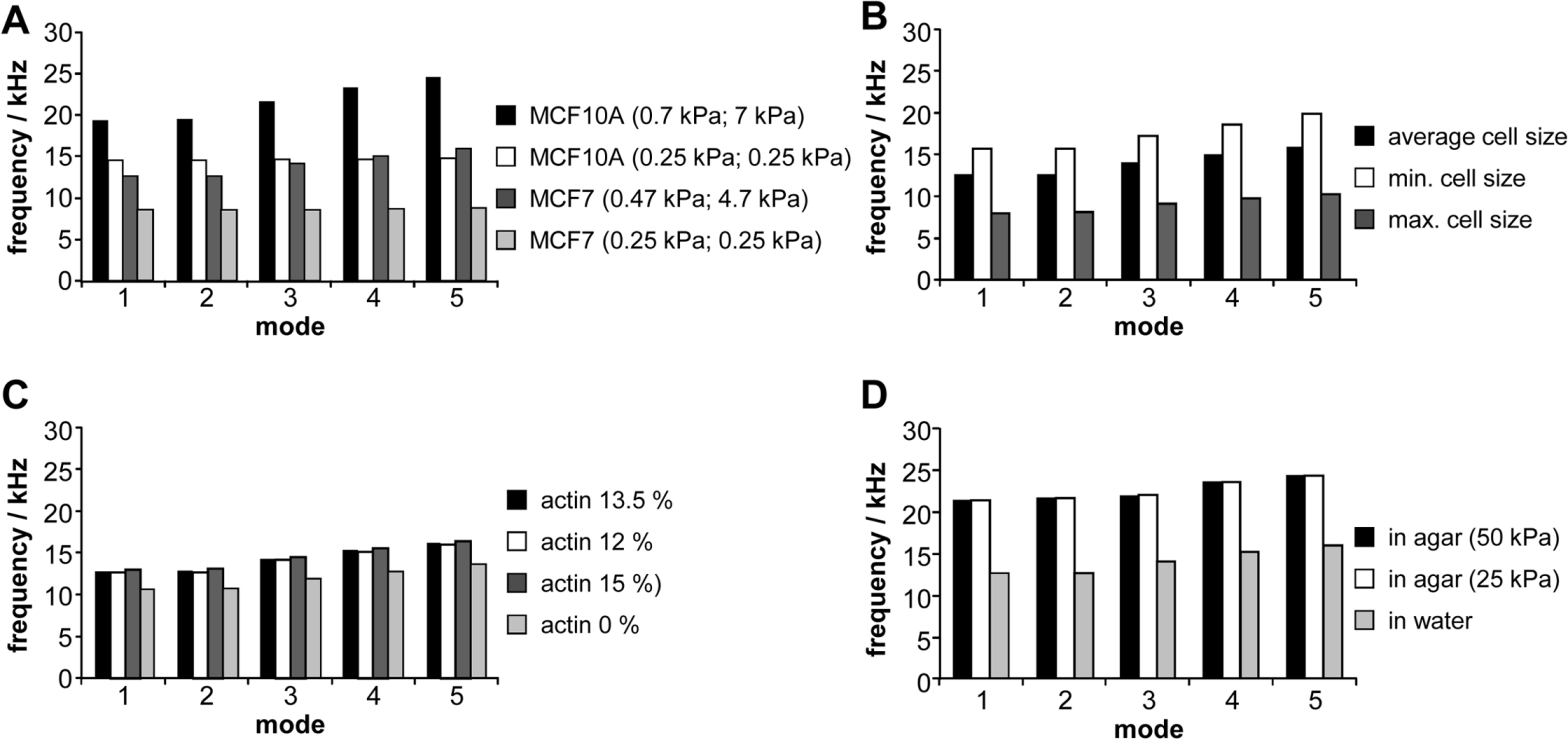
Influence of (A) material properties (Young’s modulus for cytoplasm and nucleus are as first and second value in parenthesis), (B) cell dimensions, (C) thickness of the actin cortex in percent of the cell radius, and (D) cell embedding (Young’s modulus for agar in parenthesis) on natural frequencies of MCF10A cells (A) or MCF7 cells (A-D).

With respect to the natural frequencies determined, cell dimensions, Young’s modulus of cytoplasm and nucleus as well as the embedding conditions showed the greatest influence on the shift of the natural frequencies as shown in Fig 2. The material characteristics of the nucleus and cytoplasm showed a clear influence on the natural frequencies of both cell lines (Fig 2A). The material parameters were defined according to the results of the validation. The softer model with the minimum Young’s modulus for cell plasma and nucleus reduced the resonance frequencies up to 50% for both cell types. The size of the cell also had a large impact on the level of the natural frequency (Fig 2B). Compared with the average size, the minimum cell size increased the natural frequency by up to 20%; and for the maximum cell size, the resonant frequency was reduced by up to 40%. A similar influence of cell size was also found for MCF10A cells (data not shown). The thickness of the actin cortex had only marginal effects on the natural frequencies; absence of any actin cortex caused a reduction of the resonant frequencies by 10% (Fig 2C). Compared to a cell in water, the natural frequencies for a cell embedded in an agar solution increased by 50% to 60% (Fig 2D). Reduction of the elasticity modulus of the agar solution from 50 kPa to 25 kPa led to a negligible reduction of these natural frequencies.

By comparing both cell lines, it could be concluded that the natural frequencies of the benign MCF10A cells were about 1.5 times higher than those of the malignant MCF7 cells due their smaller dimensions and higher stiffness.

### 3 Harmonic vibration analysis

**Fig 3:**
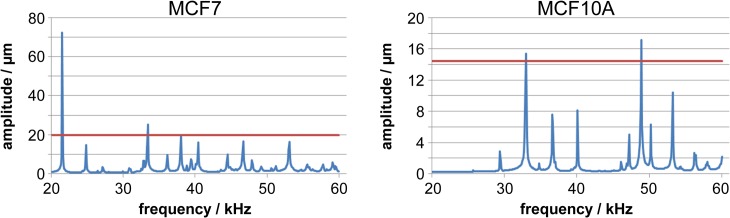
Harmonic vibration analysis of (A) MCF7 and (B) MCF10A cells (minimal sizes for both cell types) with external hydrostatic pressure in a frequency range of 20 kHz up to 60 kHz showing the displacement amplitudes. The red horizontal lines depict the maximum cell size which allows the amplitudes with the cell dimension to be compared.

As a result of a harmonic analysis, an amplitude frequency response can be determined that delivers resonance frequencies and the corresponding amplitudes. For the analysed cell models of minimum, mean, and maximum cell dimensions of both cell types, the range of excitation frequency was defined from the initial natural frequency up to 60 kHz. Cell type and dimension showed a great influence on the amplitude frequency response. A typical amplitude frequency response diagram is shown in Fig 3 for a MCF7 cells and MCF10A cells of minimum dimension (see Table 1). The depicted amplitude’s frequency response showed significant peaks at those frequencies at which oscillation forms were excited by the ultrasonic pressure. The first peak for MCF7 cells rose at 21 kHz in contrast to 34 kHz for MCF10A cells. Especially this first maximum resonance amplitude of MCF7 cells amounted to more than three times the maximum cell length, indicating huge stress on cellular integrity. The first resonance frequencies of MCF7 cells of mean dimension were also significantly lower than those of MCF10A cells. In contrast, the differences between the resonance frequencies and the amplitudes were smaller for the maximum cell dimensions of both cell lines (data not shown).

Compared to being embedded in water, the resonance frequencies for cells embedded in agar were significantly higher. The significant resonance amplitudes for the cell in agar solution were in the frequency range from 29 kHz—39 kHz (data not shown).

The influence of ultrasonic pressure and the damping coefficient on the amplitude could be considered as being linear proportional and reverse proportional, respectively. The resonance frequencies remained constant (data not shown).

### Ultrasonic irradiation of MCF7 and MCF10A cells

For experimental validation of the *in silico*-determined resonance frequencies of MCF7 and MCF10A, specific equipment for ultrasonic irradiation was constructed. An ultrasound actuator consisting of a piezoceramic and a petri dish with a duroplastic ring in its centre as reaction vessel (S1A Fig) was triggered by a function generator and an amplifier. Each ultrasound actuator was characterised for its specific frequency response in the range from 20 kHz up to 60 kHz (S1B Fig) over the whole reaction area of ~ 130 mm^2^. Since every glass petri dish possessed only certain specific resonance frequencies, a particular petri dish/actuator combination was used for each tested ultrasonic frequency.

### Selective toxicity of MCF7 cells at 24.5 kHz under 2D and 3D culture conditions

**Fig 4:**
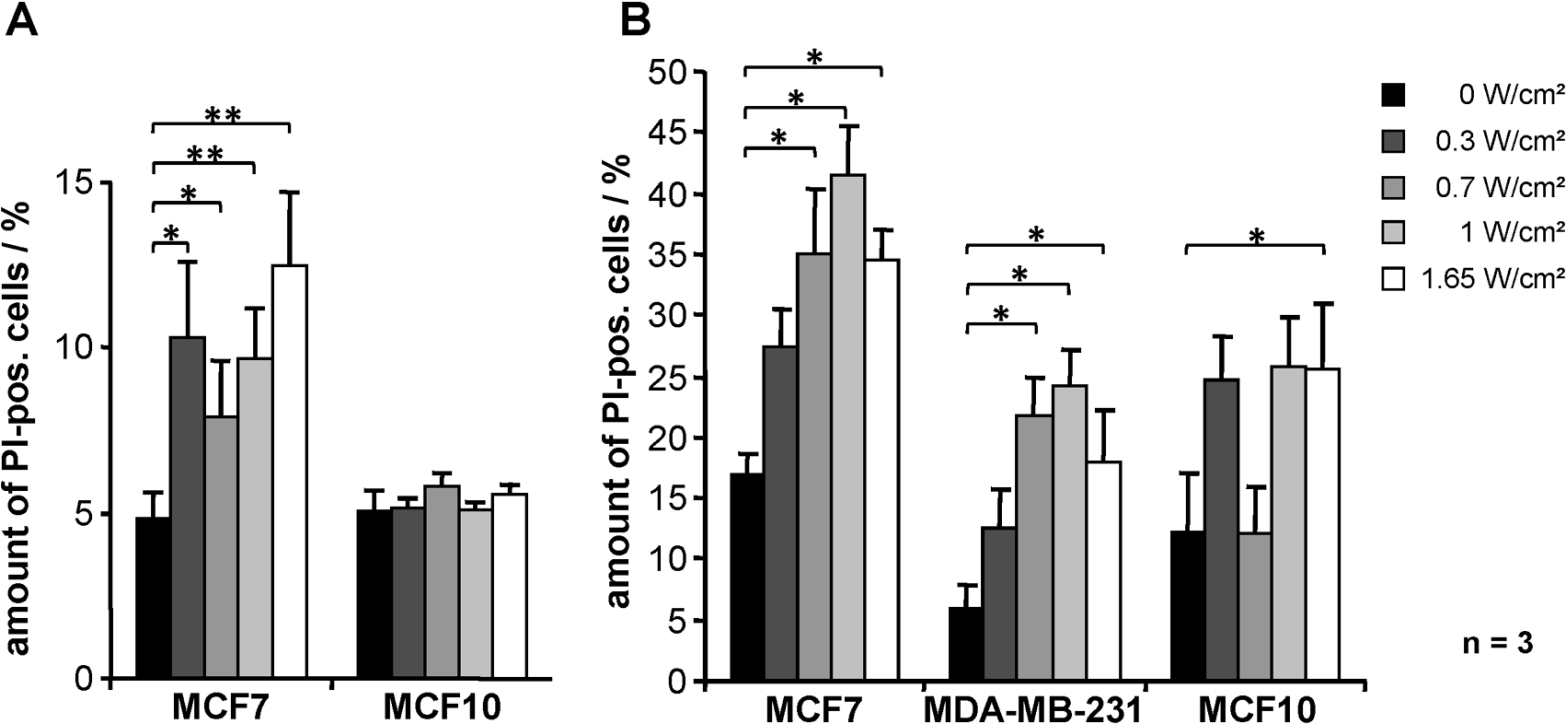
Increased death of MCF7 and MDA-MB-231 cells after irradiation with an ultrasonic frequency of 24.5 kHz. (A) Cells either cultivated in 2D culture or (B) growing in 3D culture on alginate beads (gems) were treated with 24.5 kHz and four different intensities for 4 min; 1 h later the proportion of dead cells (propidium iodide (PI) positive cells) was determined by FACS analysis. Results represent the means of data from eight (A) or three (B) independent experiments; the error bars represent the standard errors; p-values were calculated by the two-sided, paired Student’s t-test with * p<0.05, ** p<0.01.

MCF7 cells and MCF10A cells were irradiated with different ultrasonic frequencies each with four specific intensities (0.3 W/cm^2^, 0.7 W/cm^2^, 1 W/cm^2^, and 1.65 W/cm^2^). Irradiation with 24.5 kHz induced a significant increase in cell death of MCF7 cells in contrast to untreated cells (Fig 4A) resulting in up to 12.5% ± 2.2% dead cells with 1.65 W/cm^2^ (p = 0.007 vs. untreated cells). No cytotoxicity could be observed for MCF10A cells at the same frequency (Fig 4A).Treatment with 29.4 kHz or 43.6 kHz resulted in a significant increase of MCF10A cells being killed (29.4 kHz: 5.5% ± 0.8% in untreated cells to 14.7% ± 2.4% with 1 W/cm², p = 0.01; 43.6 kHz: 3.4% ± 0.8% in untreated cells to 12.4% ± 2.6% with 1 W/cm², p = 0.03) but only marginally of MCF7 cells (S2A and S2B Fig); no effect was seen in either cell line after irradiation with 51.2 kHz (S2C Fig).

In order to more accurately reflect the in vivo situation in the next setting, 3D cell growth techniques were used by culturing cells on alginate beads using the BioLevitator system. As additional cell line MDA-MB-231 (breast tumour, derived from metastatic site) was used. Treatment with 24.5 kHz again resulted in a significant increase in cell death of both malignant cell lines (Fig 4B) with a maximum after use of 1 W/cm^2^ (MCF7: 17.0% ± 1.6% in untreated cells to 41.4% ± 4.2% with 1 W/cm^2^, p = 0.02; MDA-MB-231: 6.1% ± 1.8% in untreated cells to 24.3% ± 2.8% with 1 W/cm^2^, p = 0.01). In contrast to the previous results, we also found an increase of dead MCF10A cells (12.3% ± 1.8% in untreated cells to 25.8% ± 3.9% with 1 W/cm^2^, p = 0.05).

**Fig 5:**
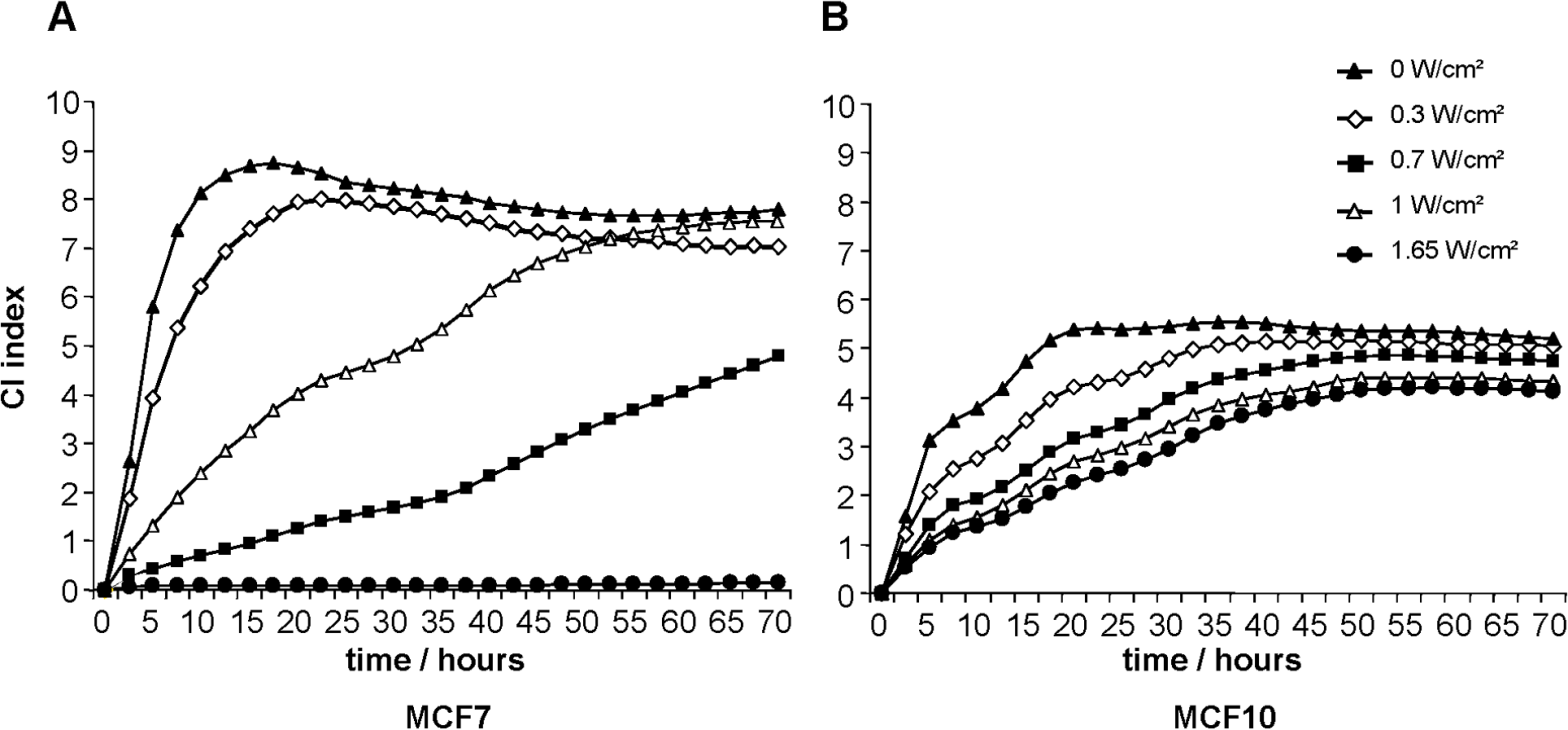
Decreased survival of MCF7 cells after irradiation with an ultrasonic frequency of 24.5 kHz as determined by XCelligence (Roche).

However, the selectivity for induction of death for MCF7 but not of MCF10A cells after treatment with 24.5 kHz was corroborated by the results using real-time and label-free xCELLigence technology. Each cell type displays its own characteristic pattern which is expressed in the Cell Index (CI). Untreated MCF7 cells reached maximal impedance after 15 hours (Fig 5A). Whereas treatment with increasing intensities resulted in a shift of the maximum to later time-points, the cells did not reach confluence with 0.7 W/cm^2^ during the observation period; after treatment with 1.65 W/cm^2^, no impedance could be measured at all, indicating that an insignificant number of cells survived ultrasonic treatment. In contrast, MCF-10 cells treated with increasing intensities and also 24.5 kHz were only delayed in proliferation, and maximal impedance values were measured in all samples (Fig 5B).

### Increased cytotoxicity of ultrasonic irradiation in brain metastases and glioblastoma cells

**Fig 6:**
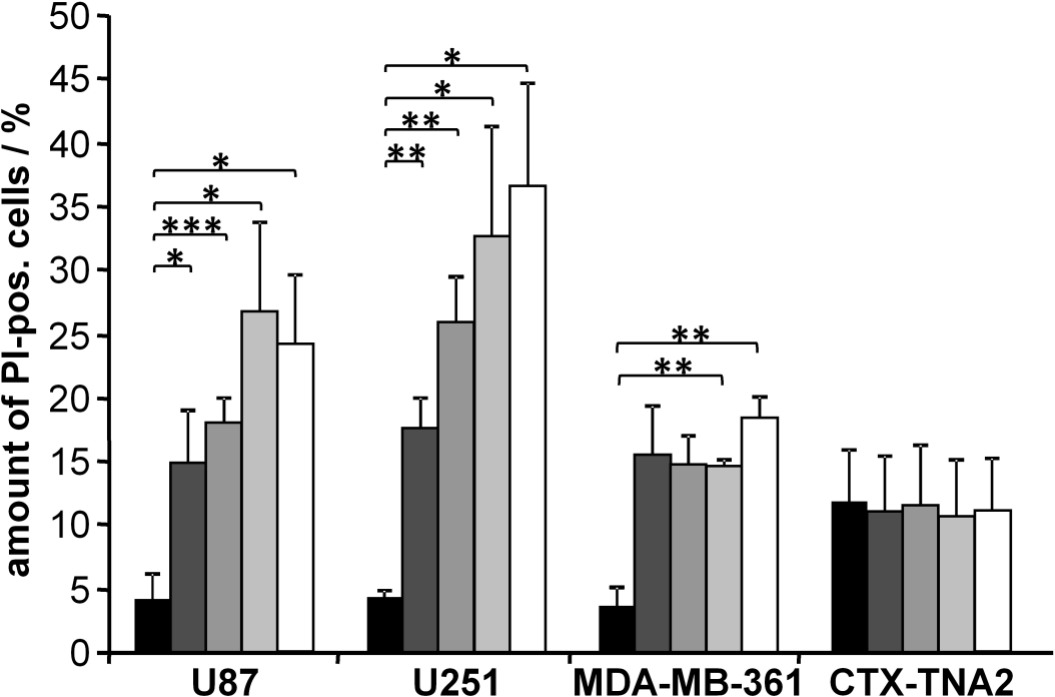
Increased death of U-87 MG, U251 MG, and MDA-MB-361 cells after irradiation with an ultrasonic frequency of 29 kHz. Normal rat astrocytes (CTX TNA2) were not affected by irradiation with 29 kHz. Results represent the means of data from four to six independent experiments; the error bars represent the standard errors; p-values were calculated by the two-sided, paired Student’s t-test with * p<0.05, ** p<0.01, *** p<0.001.

In order to broaden our findings to other cancer cell lines, we treated two glioblastoma cells lines, U-87 MG and U-251 MG, the brain metastasis cell line MDA-MB-361, and the rat astrocyte line CTX TNA2, as normal tissue cells (a human analogue has not been made commercially available) with seven different frequencies in the range of 22.9 kHz up to 51.2 kHz (data not shown). The use of 29.4 kHz resulted in a significant increase of cytotoxicity of all three tumour cell lines (Fig 6). In contrast, normal astrocytes showed no increase in cell death at this frequency (Fig 6) as well as at the other six frequencies (data not shown). Thus, we were able to show a tumour-specific ultrasonic frequency for induction of cell death.

### Increased cytotoxicity after fractionated ultrasonic irradiation

**Fig 7:**
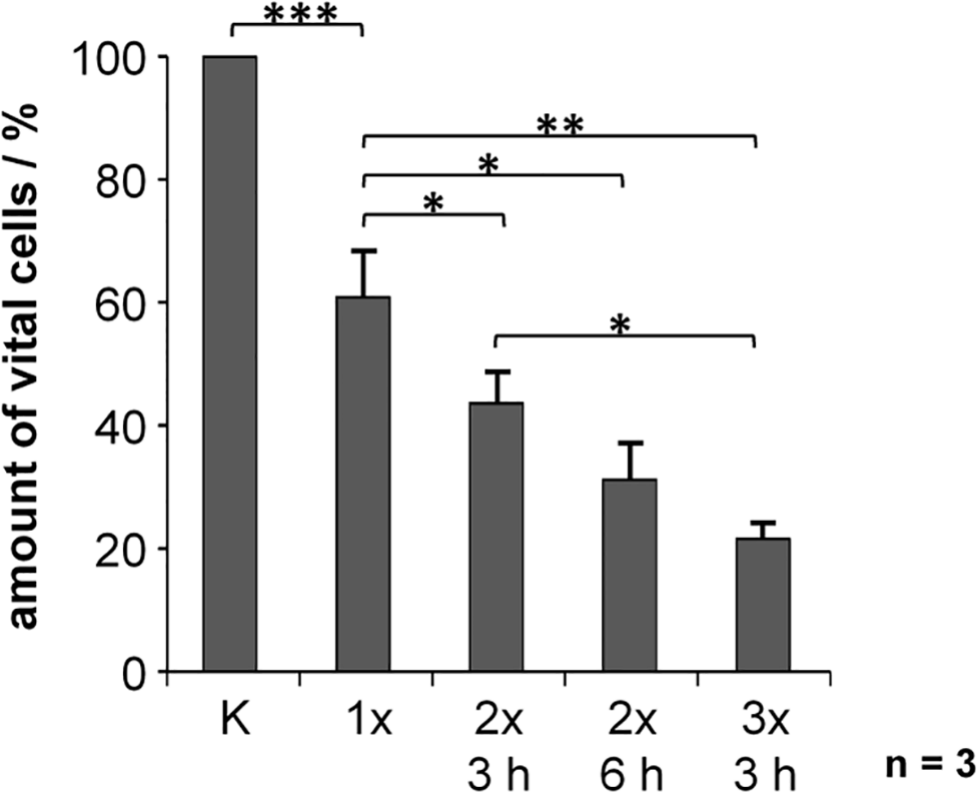
Decreased survival of HL60 cells after fractionated irradiation. HL60 suspension cells were treated by ultrasonic irradiation once, twice or three times at intervals of 3h (2x 3 h, 3x 3 h) or 6 h (2x 6 h). The number of vital cells was determined by FACS 1 h after each irradiation. (The number of vital cells of the untreated control was set as 100%.) Results represent the means of data from three independent experiments; the error bars represent the standard errors; p-values were calculated by the two-sided, paired Student’s t-test with * p<0.05, ** p<0.01, *** p<0.001.

Next, we examined if fractionated treatments by ultrasonic irradiation might result in enhanced cytotoxicity. Since repeated trypsinization of adherent MCF7 cells was not feasible, we used the suspension myeloid cell line HL60 for which we determined 24.9 kHz as the most effective frequency for cell killing. Cells were treated by ultrasonic irradiation up to three times at intervals of 3 h or 6 h. The number of viable cells was determined 1 h after each treatment. Even a singular treatment significantly reduced the number of viable cells to 60% (p = 0.0001) (Fig 7). Repeated ultrasonic irradiation resulted in a further substantial decrease of viable cells, most significant after threefold irradiation in intervals of 3 h (p = 0.02). Increasing the interval between two irradiations from 3 h to 6 h showed a trend toward increased cytotoxicity with only 43% or 31% viable cells, respectively.

### Combination of ultrasonic irradiation and paclitaxel

**Fig 8:**
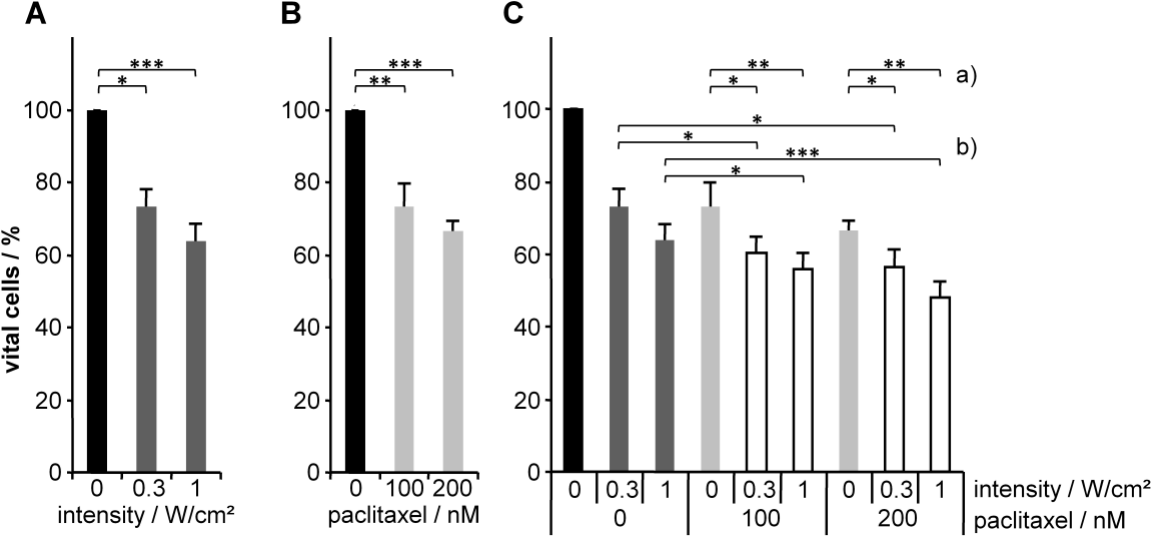
Treatment of MCF7 cells with either (A) ultrasonic irradiation with 23.22 kHz and two different intensities (0.3 W/cm^2^ or 1 W/cm^2^, dark grey bars), (B) paclitaxel with 100 nM or 200 nM (light grey bars) or (C) combinations of both treatments (ultrasonic irradiation followed by paclitaxel treatment; white bars) with a) constant concentration of paclitaxel and different intensities of ultrasonic irradiation, and b) constant intensity and different concentrations of paclitaxel. Results represent the means of data from eight independent experiments; the error bars represent the standard errors; p-values were calculated by the two-sided, paired Student’s t- test with * p<0.05, ** p<0.01, *** p<0.001.

In another experiment, we evaluated if the effect of ultrasonic irradiation might be enhanced by a combination with chemotherapy. With this in mind, MCF7 cells were treated by ultrasonic irradiation with 23.22 kHz and either 0.3 W/cm^2^ or 1 W/cm^2^ and subsequently cultivated for 48 hours with 100 nM or 200 nM of paclitaxel, one of the standard cytostatic drugs for treatment of breast cancer, (Fig 8) or else treated with paclitaxel followed by ultrasonic irradiation (S3 Fig). Monotherapy with either ultrasonic irradiation (Fig 8A) or paclitaxel (Fig 8B) resulted in a significant reduction of the numbers of vital cells to 63.9% ± 4.6% or 66.5% ± 2.8%, respectively (p = 0.0002 for 1 W/cm^2^ vs. un-irradiated control; p < 0.0001 for 200 nM vs. un-irradiated control). A further significant decrease with only 47.9% ± 4.5% of vital cells was achieved after combining ultrasonic irradiation with 1 W/cm^2^ and 200 nM paclitaxel (p = 0.0007 for combination vs. ultrasonic monotherapy; p = 0.002 for combination vs. 200 nM paclitaxel monotherapy) (Fig 8C). The results of the combination treatment in the opposite order (paclitaxel followed by ultrasonic irradiation) showed similar effects (S3 Fig). We again found a significant enhancement of the effects (reduction of proportion of surviving cells) of both monotherapies (67.1% ± 4.8% after irradiation with 1 W/cm^2^ or 69.8% ± 6.8% treatment with 200 nM paclitaxel) in their combination to only 47.9% ± 5.5% (p = 0.0003 combination vs. ultrasonic monotherapy with 1 W/cm^2^; p = 0.0008 combination vs. 200 nM paclitaxel monotherapy) (S3A–S3C Fig).

For both regimens, the comparison between monotherapy with 200 nM pactlitaxel and combination therapy with only 100 nM and ultrasonic treatment revealed a significant decrease in vital cells in the combination treatment (p = 0.02 for ultrasonic treatment (23.22 kHz, 1 W/cm^2^) + 100 nM paclitaxel; p < 0.0001 for 100 nM paclitaxel + ultrasonic treatment (23.22 kHz, 0.3 W/cm^2^); p = 0.01 for 100 nM paclitaxel + ultrasonic treatment (23.22 kHz, 1 W/cm^2^); all vs. 200 nM paclitaxel).

## Discussion

Low-frequent ultrasound was shown to selectively induce *in vitro* killing of tumour cells but not of normal tissue cells [12, 13]. Thus, we aimed to improve this technique for future application to complement standard tumour therapies. The rationale behind our study was to establish a robust scientific basis for this approach. The first part comprised *in silico* simulation by FEM analyses of the behaviour of tumour and appropriate normal tissue cells during treatment with low-frequency and low-intensity ultrasound in order to discriminate both cell types according to their resonance frequencies. In the second part, we validated these cell-specific differences *in vitro*. We focused on MCF7 and MCF10A cells because both cell lines have been extensively analysed according to their biomechanistic properties and most of the parameters we needed for detailed FEM analyses were available in various PubMed listed publications. Since different techniques were used by different groups to determine the values for specific parameters, they were partially very contradictory, especially the Young’s modulus of the nucleus ranging from 0.1 kPa up to 10 kPa and its relation to the stiffness of the cytoplasm which was described either as similar as or ten times softer than the nucleus [15, 28, 30–32]. Thus, we also considered such different parameters in our FEM simulation. We used a three-layered cell model consisting of an outer cortical actin shell, the viscoelastic cytoplasm, and the highly elastic nucleus [33]. The cellular and the nuclear membranes were not regarded as separate layers since both were shown not to be responsible for a cell’s stability [14, 34]. In contrast, it was shown that the actin shell and the lamin network determine the stability of the cell [14] or the nucleus [15, 35], respectively.

The numerical models of healthy and malignant human breast cell lines we developed were suitable to show the influence of cell dimensions, cell material properties, and cell embedding conditions on natural and excited vibration behaviour. Significant differences in natural and resonance frequencies between both cell lines were identified. Natural frequencies of the healthy MCF10A cells were found to be 1.5 times higher than the natural frequencies of the malignant MCF7 cells due to smaller dimensions and higher stiffness. As result of the harmonic analysis it was shown that the initial resonance frequencies of the MCF7 cells were lower than those of the MCF10A cells. The results of this study help to understand possible damage phenomena that can be caused by resonance amplitudes under harmonic excitation in an ultrasonic field. Other causes for cell damage under an ultrasonic field could not be investigated with these models. Due to the wide biological range of cell shapes, dimensions, and material properties in comparison to the chosen parameters for the simplified numerical models, the predicted resonance frequencies and amplitudes can only be understood as a coarse guide to show tendencies in the mechanical behaviour of human cells in an ultrasound field.

For experimental validation of the differences in sensitivity relating to low-frequency ultrasound, we were able to establish an experimental set-up for irradiation of cells with distinct ultrasonic frequencies in the range of 23 kHz to 53 kHz. Treatment of MCF7 and MCF10A cells in various 2D and even 3D culture conditions, and different analysis methods (immediate cell death but also loss of cell dividing capacity as determined by xCelligence technique) could corroborate the FEM simulation of different sensitivities of malignant and normal breast cells. This finding was further extended to MDA-MB-231 cells, a cell line established from metastatic cells in pleural effusion of a breast tumour. These malignant cells also showed increased death at a lower frequency than the MCF10A cells. This result was in line with the findings according to their elasticities [36] demonstrating that MDA-MB-231cells were significantly softer than non-malignant MCF10A cells under the same culture conditions. The Young’s modulus of MDA-MB-231 cells was shown to be 50% lower than that of MCF10A cells which is similar to the ratio of stiffness described in MCF7 and MCF10A [19]. Treatment of the glioblastoma cell lines U87 and U251 and the brain metastases cell line (derived from a primary breast tumour) MDA-MB-261 allowed a specific cytotoxic frequency of 29.4 kHz to be determined which, however, did not affect normal astrocytes. Interestingly, the rat brain-derived astrocytes used did not show any sensitivity against the ultrasonic frequencies in the range from 22.9 kHz up to 51.2 kHz. These results suggest glioblastoma as a further indication for low-frequent ultrasonic treatment, although accessibility by an appropriate actuator will be a challenge. Furthermore, fractionated ultrasonic treatment resulted in an increased rate of cytotoxicity, which also argues for fractionated application of low-frequency ultrasound during a potential clinical treatment.

A very important characteristic of the glass dish/actuator-combinations used is the non-homogenous distribution of any intensity for a specific frequency in the reaction vessel. The intensity of choice was mostly detectable in the central part accounting for about 20% of the whole reaction volume of 300 μl and was significantly lower in the border area. Thus, the partially low proportion of dead cells might be attributed to this inhomogeneous distribution of intensities and allows to speculate that increased cell death might be achieved with the treatment of a higher number of cells at the same intensity. A more homogenous launching of ultrasound in a clinical setting would be feasible by moving an ultrasonic actuator over the treatment area in tumour patients.

Although the mechanisms of induced cell death were not the focus of our studies, induction of thermal effects or of cavitation could be ruled out since a temperature increase could not be measured during ultrasonic exposure. Additionally, the intensities we used were too low for induction of cavitation [37]. In contrast, induction of specific resonance frequencies as proposed by our FEM analyses might cause a disruption of a cellular component and thus of cell death resulting in the immediate effect as early as one hour after ultrasonic irradiation. A disassembly of components of the cytoskeleton was proposed for HeLa cells after treatment with similar intensities as in our setup even though 800 kHz were applied [9]. As shown in other studies, apoptosis 12 h after treatment of U937 cells either with 1 MHz and 0.3 W/cm^2^ or 800 kHz and 1.6 W/cm^2^ [11, 38] might have contributed to the long-term effect as seen with the xCelligence.

Since new approaches in tumour therapy should synergistically complement proven concepts (e. g. chemotherapy), our results from a combination of chemotherapy and low-frequency ultrasound are very encouraging with regard to a clinical application. In a proof-of-principle experiment we clearly demonstrated that combination of low-frequency ultrasound and paclitaxel (one of the standard cytostatic drugs for treatment of breast cancer) significantly increased the cytotoxic effect of both monotherapies on MCF7 cells. Thus, we were able to prove the benefit of combination therapy of low-frequency ultrasound and chemotherapy. Especially the results of combination therapy with ultrasonic treatment and 100 nM paclitaxel, in contrast to the results of monotherapy with 200 nM paclitaxel, were most relevant with regard to a clinical use. Although the combination treatment only resulted in an additional 20% of killed cells, a regimen of fractionated ultrasonic irradiation over several days will drastically multiply this rate of tumour cell death after repeated ultrasonic treatment combined with chemotherapy compared to chemotherapy alone. A combination therapy might enable either a reduction in the duration of the chemotherapy cycles or on the other hand of the dosage of the chemotherapeutic drug.

Ultrasonic irradiation for the treatment of breast cancer might also be combined with other cytostatic drugs like 5-fluorouracil, cyclophosphamide, adriamycin, or herceptin [39, 40]. Additionally, combination therapy might also be applied during treatment of other tumour entities like malignant melanoma (ultrasound + BCNU or cisplatin [41]) or glioblastoma (ultrasound + BCNU or temozolomide [42]). Validation of combination therapy of low-frequent ultrasound plus a cytostatic drug requires further proof in a mouse model and subsequently in clinical studies.

In summary, we were able to determine for the first time for both analysed tumour cell lines a specific frequency of low-intensity ultrasound for the induction of cell ablation. For the application of this technique in tumour treatment, it is very important to consider that in contrast to the single cell level, the whole tumour is stiffer than corresponding normal tissue which was demonstrated for various tumour entities [2]. The reasons for this behaviour are due to the more compact aggregation of cells in the tumour mass than in healthy organs and an increased density of extra cellular matrix molecules [43, 44]. For example, the development of an advanced invasive mammary tumour microenvironment is accompanied with a 10- to 20-fold increase in rigidity, reaching an E-value of about 4 kPa in contrast to only 0.15 kPa of normal breast tissues [44]. In this regard our results from modelling cells in agar instead of water demonstrated a shift to about 10 kHz higher resonance frequencies (Fig 3C) and thus indicated that other frequencies will probably be required for *in vivo* application more so than *in vitro*. Furthermore, it is obvious that inhomogeneous cell populations in tumour tissues are different in their properties and are not directly comparable with the cells modelled in our FEM simulations or with the cell lines applied in our *in vitro* assays. Thus, for the treatment of different tumour types, the primary condition of an ultrasonic actor must be that it can be driven with different frequencies. The second condition is a transmittable frequency band of several kHz for an increased proportion of ablated tumour cells. Currently, there is no actor that meets these demands. The development of an ultrasonic actor operated by a variable frequency oscillator would allow the treatment of different tumour types either by direct application on the skin or by endoscopic application.

## Materials and Methods

### FEM analysis

Modelling and analysis were performed using commercial software packages Altair HyperMesh 11.0 for the mesh generation and MSC Marc/Mentat 2011, a general-purpose, non-linear FE analysis software package.

#### 1 Validation

As an initial step of generating numerical models of MCF7 and MCF10A cells for dynamic analysis, the material properties of the cell components were validated using published experimental data of an experiment with AFM (Atomic force microscopy) [19]. Adherent cell models consisting of nucleus and cytoplasm were generated for both cell lines (S4A and S4B Fig). A quasi-static numerical analysis reproducing the AFM test of Li et al. for a range of elastic properties for nucleus and plasma was carried out and the resulting force-deformation curves were compared to those experimentally determined curves for different loading rates. Considering the highly dynamic character of modal and harmonic analysis, the experimentally determined curve for the maximum loading rate was chosen as a reference curve for the definition of the material properties of the cell components.

#### 2 Modal analysis

The number of frequencies determined with a linear natural frequency analysis is given by the number of degrees of freedom of the structure, and is therefore infinite for a three-dimensional continuum. For the modal analysis of a single cell, an additional cell component was implemented. We used a three-layered model as described by Heidemann et al. with an outer cortical shell (cell membrane with underlying actin cortex), the viscoelastic cytoplasm, and the highly elastic nucleus [33]. The thickness of the shell, which was described as 17%- 23% of the diameter for normal fibroblasts and 12%- 15% of transformed fibroblasts [14], was adapted for the experimentally determined diameters of MCF10A and MCF7 cells, respectively. For endothelial cells, it was described that the actin layer amounts to a thickness of 12% to 23% of the middle cell diameter with a Young’s modulus of 1.3 to 2.8 times higher than the Young’s modulus of the plasma [45]. The Young’s modulus of MCF10A was described as 1.4 to 1.8 higher than that of MCF7 cells [19].

Different numerical models of non-adherent single cells of both cell lines were generated for a parameter study considering the biological range of cell dimensions and material properties of the cell components (S4C Fig). The cell lengths were between 14.5 μm and 26.2 μm for MCF10A and between 19.9 μm and 33.9 μm for MCF7 cells.

Two different embedding conditions were considered. For embedding in air, no fixation or additional masses acted on the outer cell surface. Embedding in water was realized by considering the hydrodynamic masses of the surrounding water between 6.9^−13^ kg and 7.3^−12^ kg for the different cell types and dimensions. Additionally, embedding of the cell in a 1% agar solution with a 2 μm-thick layer of water around the cell was studied. The first 10 natural frequencies for the biological range of geometrical and material properties for both cell lines were calculated and compared.

#### 3 Harmonic vibration analysis

In a harmonic vibration analysis performed with single cell models, the excitation due to the oscillating hydrostatic pressure from the ultrasonic field was considered. On the basis of the natural frequencies, the range of excitation frequency was defined for minimum, mean, and maximum cell size models with one characteristic material parameter set of each cell line. With the amplitude frequency response for each cell model, the resonance frequencies and the corresponding amplitudes were evaluated and compared. Furthermore, the influence of cell damping and ultrasonic pressure on the amplitude frequency response was determined. According to the experimental set up, sound intensities of 0.3 W/cm^2^ to 1.65 W/cm^2^ corresponding to sound pressures of 0.066 MPa to 0.156 MPa were used. A wide range of damping coefficients of living cells can be found in the literature [46–50]. Most of the published damping coefficients were determined for adherent cells and significantly lower frequencies than ultrasound and amount to 33% to 45%. Considering lower damping coefficients for non-adherent cells and higher frequencies, two threshold damping values of 10% and 40% were defined.

### Cell culture

All cells—MCF7, human breast cancer cell line (Sigma Aldrich, Taufkirchen, Germany); MCF10A, benign breast cell line; MDA-MB-231 (both ATCC (LGC Standards) Wesel, Germany) and MDA-MB-361 (ECACC, Sigma-Aldrich), breast cancer metastasis cell lines (pleural effusion and brain, respectively); U-87 MG and U-251 MG, glioblastoma cell lines; CTX TNA2 (ECACC, Sigma-Aldrich), established from primary cultures of type 1 astrocytes from brain frontal cortex tissue of one day old rats; and HL60, human myeloid leukaemia cell line—were cultivated at 37°C in a humidified atmosphere of 5% CO_2_; MCF7 cells in DMEM medium (Invitrogen, Karlsruhe, Germany) supplemented with 10% fetal bovine serum (FBS) (Biochrom, Berlin, Germany), MCF10A cells in DMEM/F12 (Invitrogen) containing different supplements [51], MDA-MB-231 cells in Leibovitz's L-15 medium (Biochrom) supplemented with 2 mM L-glutamine (Invitrogen) and 10% FBS, MDA-MB-361 in L15 medium supplemented with 2mM L-glutamine and 15% FBS, U-87 MG cells in DMEM supplemented with 2 mM L-glutamine and 10% FBS,CTX TNA2 cells in DMEM supplemented with 2 mM L-glutamine, 1 mM sodium pyruvate and 10% FBS, U-251 MG cells and HL60 cells in RPMI-1640 medium (Invitrogen) supplemented with 10% FBS. Diameters and volumes of MCF7 and MCF10A cells were determined with CASY Cell Count (Merck, Darmstadt, Germany). Cell cultures were periodically checked for mycoplasma by PCR. Cells were identified by Power Plex 16 system (STR-analyses, Promega, Mannheim, Germany).

### Ultrasonic actuator

For the ultrasonic irradiation of cells, a ceramic ring piezo (material: PIC151, inner diameter: 13 mm, outer diameter: 38 mm, thickness: 0.5 mm) with silver electrodes (PTYY-0153, PI Ceramic GmbH, Lederhose, Germany) was glued with epoxide resin on the bottom surface of a glass petri dish (50 mm x 12 mm, chemoLine GmbH, Hennef, Germany). As a reaction vessel, a duroplastic ring with a diameter of 13 mm (own production) was glued into the centre of the dish (S1A Fig). To drive the actuator, a sinusoidal output signal of a function generator (Agilent 33521A, Keysight Technologies GmbH, Böblingen, Germany) was boosted by an amplifier (LE 150/100 EBW, Piezomechanik GmbH, Munich, Germany) (S1C Fig). The function generator was controlled over a PC with LabView.

A critical condition for the development of a constant ultrasound wave and the vibration characteristics of the actuator is the compound of the glass, glue layer and piezo ceramic which defines specific resonance frequencies. Each petri dish/actuator combination was characterised under repeatable conditions by measuring the ultrasound intensity response over a spectrum of frequencies, ranging from 20 to 60 kHz. The intensity distribution was measured with a hydrophone (type s, RP Acoustics, Leutenbach, Germany) which was mounted on a motorized translation stage (KT70, Proxxon GmbH, Föhren, Germany). The measurement of the ultrasound intensity and the control of the translation stage was fully automated and controlled with LabView. Each actuator creates a certain number of specific narrow-band frequency peaks at each resonance frequency; usually the commonly used actuators consist of up to four resonance frequencies over a 40 kHz bandwidth (an example is shown in S1B Fig). The ultrasound intensity could be varied at each resonance frequency up to 1.65 W/cm^2^.

### Ultrasonic irradiation and determination of cell death

Cells growing in the exponential phase were harvested by trypsinization, counted, centrifuged (300x*g*) and resuspended in the appropriate medium to get a density of 3 x 10^5^ cells/ml. A volume of 300 μl was used for each 4 min irradiation. For irradiation, the glass petri dish fixed on a special mount (S1C Fig) was placed into the cell culture incubator at 37°C. 1 h after irradiation, propidium iodide (PI; stock solution with 1 mg/ml, final concentration of 10 μg/ml) was added and the proportion of vital to dead cells was analysed with FACSCalibur (BD Biosciences, San Jose, US) or guava easyCyte flow cytometer (Merck Chemicals, Schwalmbach, Germany). Data were analysed by FCS express software version 3 (DeNovo Softwae, Glendale, CA) or FlowJo (FlowJo, USA). Exemplary FACS plots are shown in S5 Fig.

For fractionated ultrasonic treatment, 4 aliquots of 300 μl HL60 cells (10^6^ cells/ml) were treated by ultrasonic irradiation for 4 min, then the 4 samples were pooled and 110 μl (sample 1) were used for cell count and FACS analysis by PI staining in parallel with untreated cells (control 1). The removed 110 μl were replaced by fresh medium. After 3 h, 600 μl cell suspension were again irradiated in two samples à 300 μl, then 110 μl of the treated 600 μl (sample 2) and 110 μl of the untreated cells (sample 3) were used for cell count and FACS analysis in parallel with completely untreated cells (control 2). After 6 h, 300 μl of the cells that had been irradiated twice (after 0 h and 3 h, sample 4) and 300 μl of the cells that had been irradiated at the beginning (after 0 h, sample 5) were again irradiated. For all samples (un-irradiated at all time-points and irradiated after 6 h), 110 μl were used for cell count and FACS analysis.

### Microcarrier cell culture

For the establishment of a three dimensional model, the bench-top bioreactor BioLevitator (Hamilton Company, Switzerland) and basal membrane coated GEM were used. Cells were incubated in specialized culture vessels (LeviTubes; Hamilton)) in the bioreactor at 37°C and 5% CO2. 3×10^6^ cells were seeded on 1 ml GEM pre-coated with collagen IV or gelatine (Omni Life Science, Bremen, Germany) in a total volume of 10 ml medium with 10% FBS. After an overnight inoculation period, LeviTubes were filled with an additional 15 ml of medium. An aliquot of the microcarrier cultures was treated with ultrasonic irradiation, 1 h later GEM were dissolved in pre-warmed trypsin/EDTA, GEM were removed by a magnet, the restraining cells were stained with PI measurement and analysed by FACSCalibur (BD Biosciences, San Jose, US) and by FCSexpress De Novo (Glendale, CA) as described above. Staining was additionally documented on a LSM510 Meta confocal laser scanning microscope (Zeiss, Germany).

### xCELLigence system

The xCELLigence DP device from Roche Diagnostics (Mannheim, Germany) was used to monitor cell proliferation in real-time. This system allows the measurement of the impedance in real-time; the higher the value of the impedance, the higher the amount of vital cells growing on the surface of each well; a maximal value is reached when the cell layer becomes confluent. After ultrasonic irradiation with 24.5 kHz, 5 × 10^3^ or 1 × 10^4^ cells were seeded per well in quadruplicates in electronic microtiter plates (E-Plate; Roche Diagnostic) and measured with programmed signal detection every 20 min for 92 h with the xCELLigence system according to the manufacturer’s instructions. Data acquisition and analyses were performed with the RTCA software (version 1.2, Roche Diagnostics).

### MTT-assay

Subsequent to ultrasonic irradiation, 5000 cells in medium without paclitaxel or medium containing 100 nM or 200 nM paclitaxel (Bristol-Myers Squibb, Munich, Germany) were seeded per well of a 96-well plate. 48 h later the MTT assay was performed as described previously [52].

### Statistical analysis

As indicated in figure legends, results are presented as means ± standard error and represent at least three independent experiments. P-values (calculated by the two-sided, paired student’s t-test) less than or equal to the significance level 0.05 were designated as statistically significant.

## Supporting Information

S1 Fig (A) A glass petri dish containing a ceramic ring piezo on the lower surface and a duroplastic ring as reaction vessel was used for ultrasonic irradiation of cells in suspension. (B) A sinusoidal output signal generated by a function generator (a) and boosted by an amplifier (b) was used to drive the ultrasonic actuator fixed on a specific mount (c). (C) An example of a typical ultrasound intensity response curve for such a petri dish/actuator combination of a range of 20 to 60 kHz showing two specific narrow-band frequency peaks at 46 kHz and 53 kHz. (TIF)

S2 Fig Difference in responsiveness of MCF7, MCF10A, and MDA-MB-231 cells to different ultrasonic frequencies. Cells in suspension were treated with ultrasonic frequencies of (A) 29.4 kHz, (B) 43.6 kHz, or (C) 51.2 kHz each with four different intensities. 1 h later the number of dead cells (propidium iodide (PI) positive cells) was determined by FACS analysis. Results represent the means of data from six independent experiments; the error bars represent the standard errors; p-values were calculated by the two-sided, paired Student’s t-test with * p<0.05, *** p<0.001.
(TIF)

S3 Fig Treatment of MCF7 cells with either (A) ultrasonic irradiation with 23.22 kHz and two different intensities (0.3 W/cm^2^ or 1 W/cm^2^, dark grey bars), (B) paclitaxel with 100 nM or 200 nM (light grey bars) or (C) combinations of both treatments (paclitaxel treatment followed by ultrasonic irradiation; white bars) with a) constant concentration of paclitaxel and different intensities of ultrasonic irradiation, and b) constant intensity and different concentrations of paclitaxel. Results represent the means of data from seven independent experiments; the error bars represent the standard errors; p-values were calculated by the two-sided, paired Student’s t- test with * p<0.05, ** p<0.01, *** p<0.001.
(TIF)

S4 Fig (A) Three-dimensional numerical grid model of an adherent cell. (B) Setup for numerical analysis of AFM-test (red: nucleus, green: cytoplasma). Arrow and circle above the nucleus signify the pressure on a cell by i. e. the cantilever during AFM analysis. (C) Numerical model of MCF10A cell with actin layer 20% (cutting view). (TIF)

S5 Fig FACS measurements from representative experiments. The percentage of PI fluorescence signal of MCF7, MCF10A, or MDA-MB-231 cells cultured under 2D (A) or 3D (B) conditions and either left untreated (0 W/cm2) or were treated with 24 kHz and specific intensities (0.3 W/cm2, 0.7 W/cm2 1 W/cm2 and 1.65 W/cm2) are shown. Small non-definable population was only visible by irradiated MCF7 cells, marked with an arrow and increased by the treatment.
(TIF)
