# Supplementary figures and images for: Characterization of Dynamic Behaviour of MCF7 and MCF10A Cells in Ultrasonic Field Using Modal and Harmonic Analyses
Source: PLoS One. 2015 Aug 4;10(8):e0134999. doi: 10.1371/journal.pone.0134999 (PMC4524665; doi:10.1371/journal.pone.0134999)

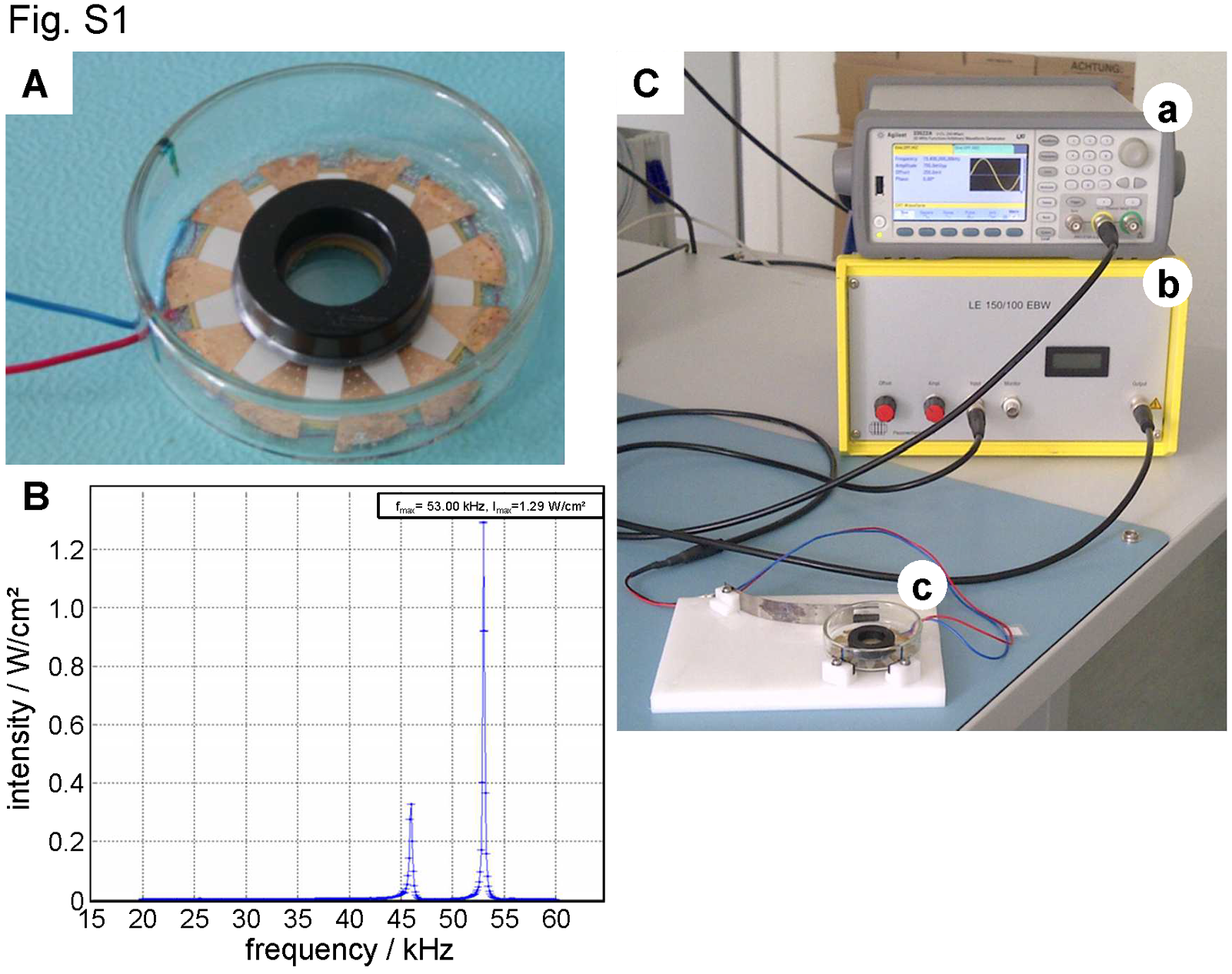

Supplement: S1 Fig — (TIF) [file pone.0134999.s001.tif]

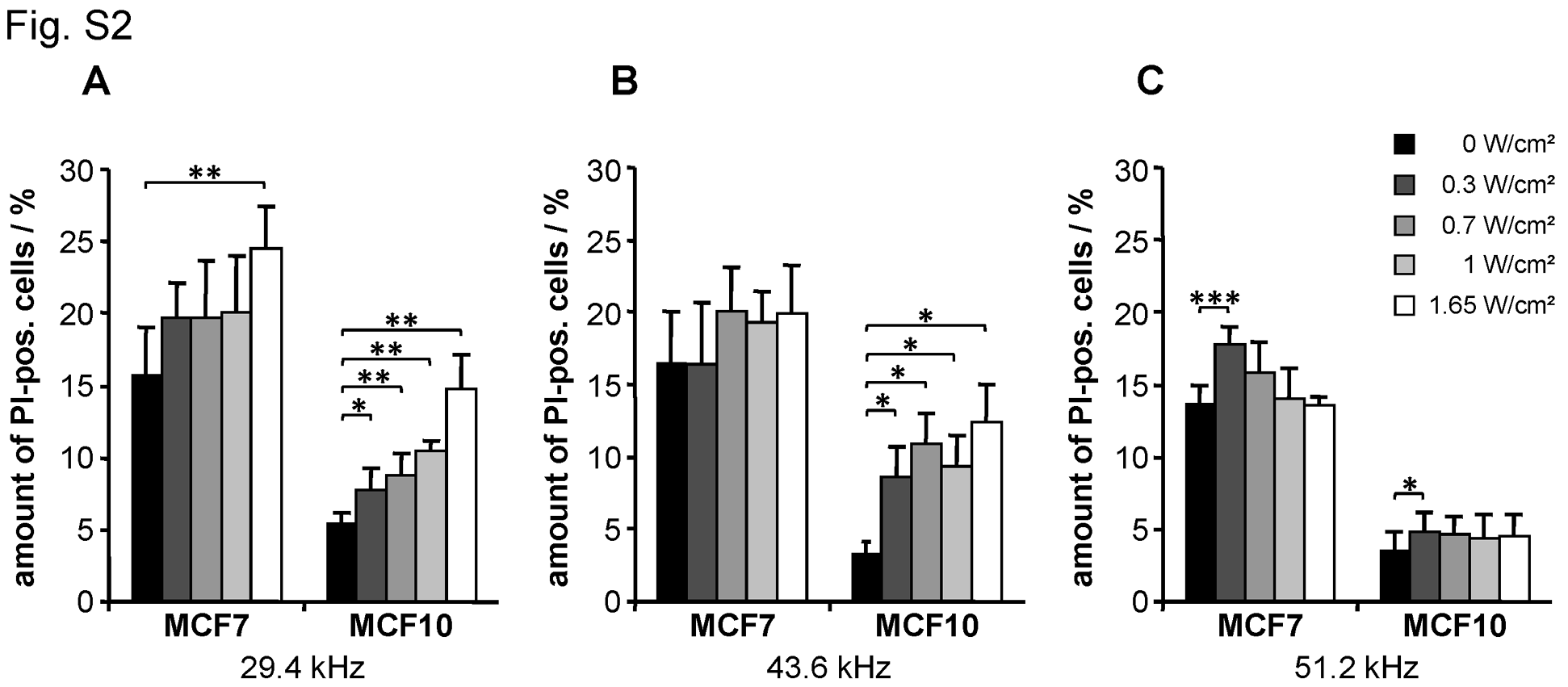

Supplement: S2 Fig — Cells in suspension were treated with ultrasonic frequencies of (A) 29.4 kHz, (B) 43.6 kHz, or (C) 51.2 kHz each with four different intensities. 1 h later the number of dead cells (propidium iodide (PI) positive cells) was determined by FACS analysis. Results represent the means of data from six independent experiments; the error bars represent the standard errors; p-values were calculated by the two-sided, paired Student’s t-test with * p<0.05, *** p<0.001. (TIF) [file pone.0134999.s002.tif]

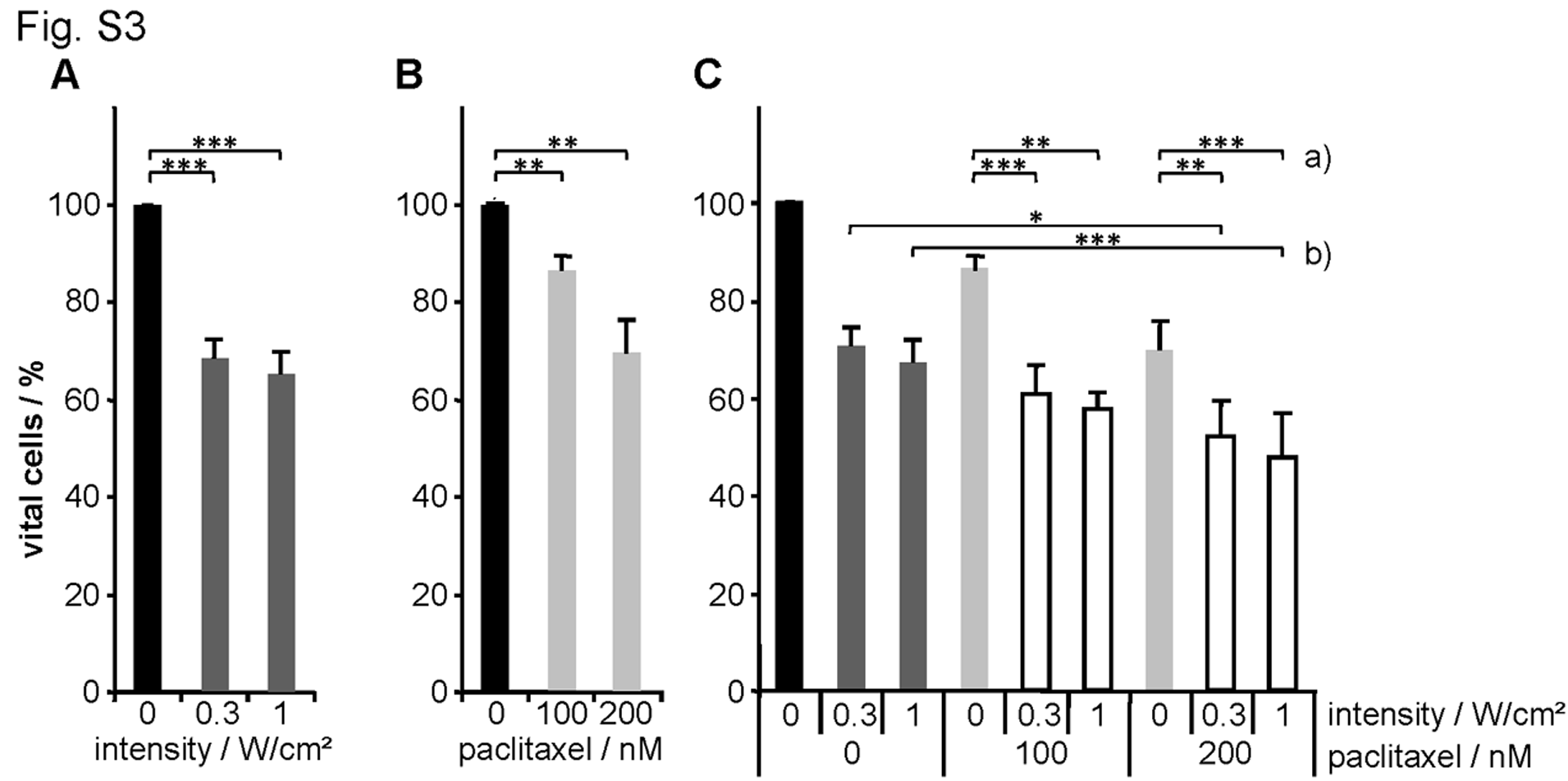

Supplement: S3 Fig — Results represent the means of data from seven independent experiments; the error bars represent the standard errors; p-values were calculated by the two-sided, paired Student’s t- test with * p<0.05, ** p<0.01, *** p<0.001. (TIF) [file pone.0134999.s003.tif]

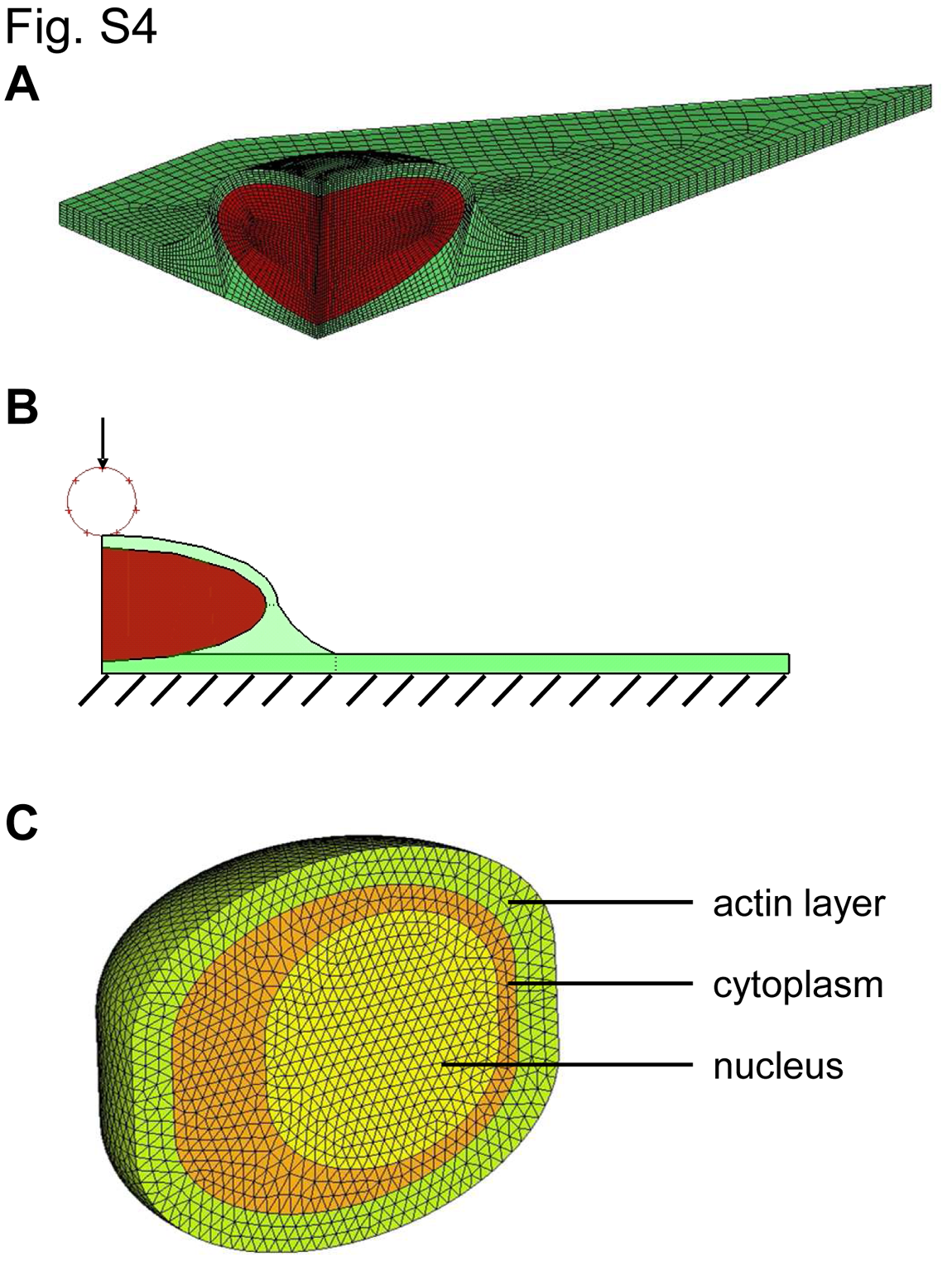

Supplement: S4 Fig — (TIF) [file pone.0134999.s004.tif]

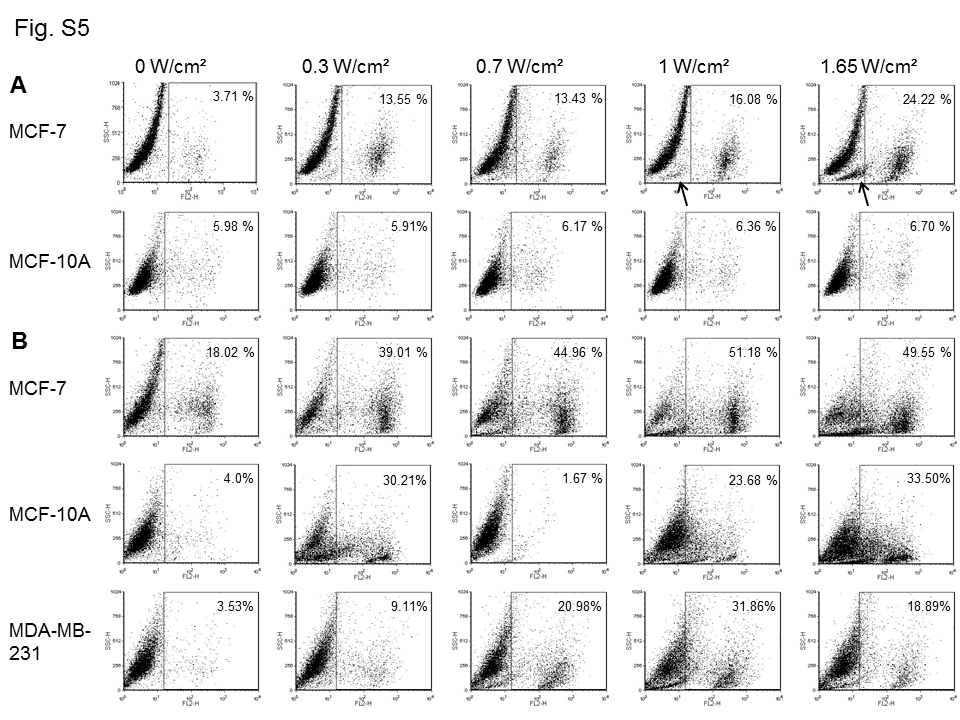

Supplement: S5 Fig — The percentage of PI fluorescence signal of MCF7, MCF10A, or MDA-MB-231 cells cultured under 2D (A) or 3D (B) conditions and either left untreated (0 W/cm2) or were treated with 24 kHz and specific intensities (0.3 W/cm2, 0.7 W/cm2 1 W/cm2 and 1.65 W/cm2) are shown. Small non-definable population was only visible by irradiated MCF7 cells, marked with an arrow and increased by the treatment. (TIF) [file pone.0134999.s005.tif]
